# Evaluación de la calidad de vida en niños y adolescentes con diabetes de tipo 1 en dos instituciones de salud, Bogotá, D. C., Colombia

**DOI:** 10.7705/biomedica.6675

**Published:** 2023-03-30

**Authors:** María Isabel García, Camila Céspedes, Paola Durán, Mauricio Coll, Catalina Forero

**Affiliations:** 1 Hospital Universitario San Ignacio, Pontificia Universidad Javeriana, Bogotá, D. C., Colombia Pontificia Universidad Javeriana Hospital Universitario San Ignacio, Pontificia Universidad Javeriana Bogotá Colombia; 2 Centro de Endocrinología Pediátrica y del Adolescente Endociencia, Bogotá, D. C., Colombia Centro de Endocrinología Pediátrica y del Adolescente Endociencia Bogotá Colombia

**Keywords:** diabetes mellitus de tipo 1, calidad de vida, niño, adolescente, Type 1 diabetes mellitus, quality of life, child, adolescent

## Abstract

**Introducción.:**

La diabetes mellitus es una de las enfermedades crónicas con mayor prevalencia en la población pediátrica y juvenil, con efectos en la calidad de vida de los pacientes.

**Objetivo.:**

Evaluar la calidad de vida de una población pediátrica menor de 18 años con diagnóstico de diabetes de tipo 1, de dos instituciones pediátricas de la ciudad de Bogotá.

**Materiales y métodos.:**

Se recolectaron los datos sociodemográficos, y se emplearon la versión validada en español del cuestionario PedsQL4.0™ y el módulo 3.2 sobre diabetes. Los datos se procesaron en el *software* estadístico STATA 17™.

**Resultados.:**

Con el puntaje global del módulo 3.2 sobre diabetes, de la versión validada del PedsQL™, se evaluó la correlación entre los valores de la hemoglobina A1c (HbA1c) y los del cuestionario. Los pacientes con valores por debajo del 9 % de HbA1c presentaron una mejor calidad de vida relacionada con la salud, mientras que, en el grupo con HbA1c mayor de 9 %, se observó una baja percepción de calidad de vida (p=0,025). En cuanto el tipo de terapia y la relación con los dominios del PedsQL™ 3.2, versión diabetes, los pacientes que utilizaban la bomba de insulina o microinfusor presentaban mejor puntaje en los dominios barreras, cumplimiento, preocupación y comunicación, y en el puntaje global, respecto a quienes usaban múltiples inyecciones de insulina como tratamiento (p=0,0363).

**Conclusiones.:**

En nuestros pacientes, un mejor control metabólico (medido por el valor de HbA1 c) y el uso de microinfusora contribuyen aúna percepción de mejor calidad de vida.

## Abstract (en)

**Introduction:**

Diabetes mellitus is one of the most prevalent chronic diseases in the pediatric and juvenile population that affects the quality of life of patients.

**Objective:**

To evaluate the quality of life of a pediatric population under 18 years of age diagnosed with type 1 diabetes from two pediatric institutions in the city of Bogotá.

**Material and methods:**

We collected of sociodemographic data and clinical variables and application of the PedsQL 4.0™ questionnaire, and the diabetes module 3.2 version validated in Spanish. The sociodemographic data, the clinical variables and the PedsQL™ were processed in the statistical software Stata 17™.

**Results:**

In the global score of the PedsQL™ 3.2, diabetes version, men presented better quality of life compared to women. The correlation between the hemoglobin Ale (HbA1c) values and the PedsQL scale in the global score was evaluated. Patients with HbA1c values below 9% presented a better health-related quality of life, while in the group with HbA1c greater than 9% a perception of low quality of life was observed (p-0.025).

Regarding the type of therapy and the relationship with the domains of the PedsQL 3.2, diabetes version, patients who used insulin pumps had better scores in the domains barriers, adherence, concern, communication and in the global score compared to patients who used multiple daily injections of insulin as treatment (p-0.0363).

**Conclusions:**

in our patients, a better metabolic control (measured by the HbA1c value) and the use of an insulin pump contribute to a better perception of quality of life.

La diabetes mellitus es una de las enfermedades crónicas con mayor prevalencia en la población juvenil y pediátrica. Se estima que la tasa de afectados menores de 15 años ronda entre el 0,3 % a nivel mundial y el 0,15 % para los países desarrollados ^1^. Según el proyecto DiaMond (*Diabetes Mondiale*) de la Organización Mundial de la Salud (OMS), con el cual se evalúa la incidencia de la diabetes en el mundo, en lo que corresponde a Latinoamérica, Perú, Bolivia y Paraguay tienen las tasas más bajas, dado que cuentan con un gran porcentaje de población indígena, lo cual, al parecer, actúa como factor protector. Le siguen algunos países de Centroamérica con la misma concentración de población indígena con baja incidencia. Por otra parte, Chile y Brasil cuentan con tasas de incidencia más altas, actualmente, del 6,5 al 8,6 por 100.000 habitantes; le siguen Argentina y Uruguay, con una incidencia del 8 por 100.000 habitantes. Si se agregan Canadá y Estados Unidos, para tener toda América, Canadá es el que tiene la tasa de incidencia más alta, casi 23 por 100.000. En Colombia, la incidencia reportada es de 3 a 4 casos anuales por cada 100.000 niños menores de 15 años ^2^.

Los datos más recientes sobre incidencia en menores de 15 años de diabetes de tipo 1 en Colombia, fueron reportados I en 5,3 por cada 100.000 niños para Bogotá en el año 2008 ^3^. Según las estadísticas de la Asociación Colombiana de Diabetes, al año se diagnostican 55 a 60 menores de edad, en promedio. Es una enfermedad de alto costo para el sistema de salud, por lo cual es fundamental un manejo adecuado.

La diabetes mellitus de tipo 1 produce complicaciones tanto agudas (hipoglucemia, cetoacidosis diabética) como crónicas (alteraciones microvasculares y macrovasculares); además, genera implicaciones psicológicas, emocionales y sociales. Por tanto, el tratamiento de la diabetes en pacientes pediátricos debe abordarse desde una óptica dirigida, no solo a controlar los síntomas directos y objetivos de la enfermedad, sino también, a optimizar todos los aspectos inherentes a su bienestar y calidad de vida ^4^.

La noción de calidad de vida es una ¡dea que nació en la OMS en 1994; no obstante, no fue sino hasta épocas recientes que se incorporó, en el ámbito de la salud, el concepto de calidad de vida relacionada con la salud. Las diferentes escalas de medición epidemiológica han demostrado que el tratamiento de diversas enfermedades debería incluir aspectos como el estado de ánimo o el soporte social que se le ofrece al paciente, entre otros elementos dirigidos a fortalecer la percepción de su propio bienestar ^5^.

En la actualidad, existe un considerable interés por evaluar la calidad de vida de los pacientes diabéticos en forma integral, considerando los aspectos físicos propios de la enfermedad y, también, los elementos que forman parte de su cotidianidad. Esto parte del principio de que la calidad de vida y el bienestar integral de estos sujetos cumplen efectivamente un rol fundamental en el control metabólico y la concentración de hemoglobina glucosilada.

En Colombia, existen datos sobre la calidad de vida relacionada con la salud en pacientes adultos con diabetes de tipo 2. Sin embargo, no se cuenta con información de la misma en pacientes adultos ni pediátricos con diabetes de tipo 1, por lo cual el objetivo de este estudio fue obtener datos nacionales sobre la calidad de vida relacionada con la salud, con el fin de, además de caracterizarla, poder construir propuestas y estrategias para evaluarla, hacerle seguimiento y mejorarla.

## Materiales y métodos

Se trata de un estudio observacional y descriptivo de corte transversal, realizado con los pacientes diabéticos de las consultas de endocrinología pediátrica del Hospital San Ignacio y Endociencia, de Bogotá. Se incluyeron los pacientes entre 2 y 18 años para la fecha de la encuesta, con diagnóstico de diabetes de tipo 1 y con un tiempo de evolución de la enfermedad mayor de 6 meses; además, se incluyeron los padres o acudientes que contaran con alfabetización digital.

Se excluyeron aquellos con alteraciones sensoriales o cognitivas diagnosticadas, discapacidad física conocida e inicio de la diabetes mellitus. En un primer contacto telefónico, se recolectaron datos referentes a la evolución de la diabetes, tales como antecedentes familiares y personales, y diferentes aspectos sociales relacionados.

Posteriormente, se valoró la calidad de vida relacionada con la salud por medio de correo electrónico, entrevista telefónica o ambos, previa firma de un consentimiento informado. La entrevista fue atendida por los propios niños cuando eran mayores de 8 años y, por los padres, en los menores de esa edad.

La herramienta utilizada para las entrevistas fue el cuestionario validado en español PedsQL™ 3.2, módulo para diabetes. El *Pediatric Quality of Life Inventory™* (PedsQL™) (https://www.pedsql.org) es un instrumento modular para medir la calidad de vida relacionada con la salud en niños y adolescentes de 2 a 18 años. Comprende un cuestionario general sobre salud (módulo PedsQL 4.0™) y otro complementario específico de la enfermedad que se estudie, en este caso, el módulo para diabetes (PedsQL™3.2), también dirigido a padres y niños. Es un instrumento validado, de fácil y rápida aplicabilidad, y estructurado por grupos etarios. Los padres o acudientes responden las versiones del cuestionario en casos de menores de 8 años.

El módulo para diabetes 3.2 del PedsQL™ es el cuestionario validado traducido al español. Consta de 33 preguntas organizadas en cinco módulos: el primero, con 15 preguntas relacionadas con los síntomas de la diabetes; el segundo, con 5 preguntas que valoran las posibles barreras ante el tratamiento; el tercero, con 6 preguntas relativas al cumplimiento del tratamiento; el cuarto, con 3 preguntas que indagan sobre la preocupación por posibles complicaciones, tanto agudas como futuras, y el quinto, con las 4 últimas preguntas que valoran la comunicación con los demás respecto al tema de la diabetes, incluyendo la relación con los profesionales de la salud.

Cada pregunta tiene cinco opciones de respuesta acorde con la percepción del paciente, la de sus padres o la de ambos. Las respuestas obtenidas se convierten a una escala de 0 a 100. Los valores más altos indican mejor calidad de vida. Los datos fueron recogidos por medio del *software* REDCap (*Research Electronic Data Capture*) y, posteriormente, se efectuaron los análisis estadísticos con el programa de Stata 17™.

Para las variables cuantitativas, si presentaban distribución normal, se calcularon promedios y desviaciones estándar, y, en caso contrario, se calcularon medianas y rangos intercuartílicos. Para las variables cualitativas, se estimaron frecuencias absolutas y proporciones.

El análisis de la calidad de vida global y por dominios se hizo de forma general para toda la población y por subgrupos de edad, sexo, tiempo de diagnóstico de la enfermedad, control metabólico, regularidad en el seguimiento por endocrinología pediátrica y tipo de terapia, para lo cual se compararon las medianas entre los grupos previamente descritos. Para las variables hasta de dos categorías, se empleó la prueba U de Mann-Whitney y, para las variables de más de dos categorías, se empleó Kruskal-Wallis. A pesar de que la muestra no era aleatoria, se calcularon los valores de p, para evidenciar dónde podría haber diferencias, plantear estudios y probar futuras hipótesis.

## Resultados

### Características sociodemográficas

Se contactaron 32 pacientes del Hospital Universitario San Ignacio y de Endociencia mediante sus médicos tratantes, de los cuales 25 aceptaron y firmaron el consentimiento informado para el estudio: 17 (68 %) pacientes del Hospital San Ignacio y 8 (32 %) de Endociencia.

El 72 % eran hombres, la edad media en el momento del estudio fue de 13,2 años (rango: 2,15-17,5 años) y el 96 % se encontraban escolarizados; la mayoría de los pacientes pertenecían al estrato socioeconómico 3 (cuadro 1).

**Cuadro 1:** Características sociodemográficas de los 25 pacientes incluidos en el estudio (n=25)

Edad (años)		13,21 (2,15-17,5) DE=3,79
Sexo (n, %)		
	Masculino	18(72)
	Femenino	7(28)
Escolaridad (n, %)		
	No escolarizado	1 (4)
	Preescolar	4(16)
	Básica primaria	16 (64)
	Básica secundaria	4(16)
Estrato socioeconómico (n, %)		
	1	1 (4)
	2	5(20)
	3	11 (44)
	4	1 (4)
	5	5(20)
	6	2(8)

DE: desviación estándar

El tiempo promedio de evolución de la enfermedad fue de 4,39 años (rango: 0,5-14 años), con una desviación estándar (DE) de 3,04, y la media de la hemoglobina A1c (HbA1c) de este grupo fue de 8,16 % (rango: 5,5-12 %), con una DE de 1,67 %.

El 76 % de los pacientes eran eutróficos y el 16 % tenían sobrepeso u obesidad, según las curvas de índice de masa corporal de los *Centers for Disease Control and Prevention* (CDC).

El 80 % de los pacientes tenía el esquema de múltiples inyecciones diarias, y el 20 % restante usaba microinfusores de insulina. Todos los pacientes se autocontrolaban, el 40 % mediante glucometría capilar, el 40 % con monitoreo *flash* intermitente de glucosa y el 20 % con un monitor acoplado a la microinfusora. El promedio de tiempo desde el último control por endocrinología pediátrica fue de 2,6 meses (rango: 1 -7 meses), con una desviación estándar (DE) de 1,41.

Solo en 8 (32 %) pacientes de 25, la hemoglobina glucosilada (HbA1c) fue menor de 7 %; en 7 (28 %), estuvo entre 7,01 y 9 % (n 7), y en los 10 (40 %) restantes, sus valores fueron mayores de 9 %.

El 88 % no tenían comorbilidades y, entre quienes las reportaron, las más frecuentes fueron hipotiroidismo y asma. No se encontraron complicaciones macrovasculares o microvasculares, ni dislipidemia.

### Resultados del cuestionario PedsQL™3.2

La calidad de vida relacionada con la salud se consideró deficiente como una puntuación autoinformada por el niño de 70 o menor en el PedsQL™, o una puntuación de 65 o menor informada por el padre o acudiente. Los puntos de corte se eligieron con base en un estudio de Varni, *et al.,* del 2003 ^6^.

Se compararon diferentes variables independientes. En el cuadro 2, se muestra la comparación entre los diferentes dominios de la encuesta y el sexo, encontrándose que los hombres referían sentir más síntomas que las mujeres. El promedio mínimo de calidad de vida relacionada con la salud para ambos sexos fue de 31,6, con un promedio máximo de 93,3 y una media de 73. Los valores máximos indican una mejor percepción de los síntomas de diabetes. En los dominios de barreras, preocupación, cumplimiento y comunicación, las mujeres presentaron puntajes inferiores a los de los hombres. En cuanto al puntaje global del PedsQL™ 3.2 sobre diabetes, los hombres presentaron mejor calidad de vida que las mujeres. Ninguna de las diferencias encontradas fue estadísticamente significativa (cuadro 2).

**Cuadro 2:** Comparación de dominios del PedsQL™ 3.2, módulo sobre diabetes, *Vs.* sexo y hemoglobina glucosilada

Dominio	Hombres	Mujeres	p	<7%	7,01-9%	>9%	p
Síntomas	70,8 (18,3)	73,3 (25)	0,9188	79,1 (20,8)	70 (20)	70 (18,3)	0,247
Barreras	65 (30)	50 (25)	0,1281	70 (37,5)	65 (20)	42,5 (30)	0,068
Cumplimiento	83,3 (29,2)	79,2 (33,3)	0,7992	87,5 (27)	91,6 (20,8)	66,6 (25)	0,086
Preocupación	66,7 (58,3)	41,7 (41,6)	0,3969	70,8 (33,3)	66,6 (75)	37,5 (58,3)	0,099
Comunicación	93,7 (18,7)	87,5 (37,5)	0,4973	93,7 (6,25)	100 (6,25)	84,3 (56,2)	0,200
Puntaje global	72 (20)	62,8 (27,2)	0,1550	81,75 (21,2)	74,08 (20)	61,3 (21,3)	0,025

* Se describen medianas y, entre paréntesis, los rangos intercuartílicos.

Se comparó el tipo de tratamiento y la relación con los dominios del PedsQL™ 3.2 (versión para diabetes). Los pacientes usuarios de una bomba microinfusora de insulina presentaron mejor puntaje en los dominios de barreras, cumplimiento, preocupación y comunicación, y en el puntaje global respecto a los pacientes empleaban múltiples inyecciones de insulina como tratamiento. El único dominio en que no se evidenció esta tendencia fue el de síntomas. La relación del tipo de terapia con el puntaje global de calidad de vida en diabetes fue estadísticamente significativa (cuadro 3).

**Cuadro 3:** Comparación de los dominios del PedsQL™, versión 3.2, módulo sobre diabetes, y tipo de tratamiento

Dominio	Múltiples inyecciones	Microinfusora	p
Síntomas	74,1 (22,5)	70 (3,33)	0,7592
Barreras	57,5 (27,5)	75 (25)	0,1251
Cumplimiento	81,25 (29,1)	87,5 (20,8)	0,5611
Preocupación	45,8 (54,1)	66,6 (33,3)	0,0705
Comunicación	90,6 (15,6)	100 (0)	0,0653
Puntaje global	66,2 (20)	70 (18,3)	0,0363

* Se describen medianas y, entre paréntesis, los rangos intercuartílicos.

Se evaluó la correlación entre los valores de hemoglobina glucosilada y la escala PedsQL™, y se encontraron buenos puntajes en los dominios de síntomas y comunicación para todos los valores de HbA1c, aunque sin diferencias estadísticamente significativas. En el grupo de HbA1c mayor de 9 %, se documentaron puntajes más bajos en barreras, cumplimiento y preocupación, mientras que, en el grupo con HbA1c menor de 7 %, todos los dominios presentaban puntajes altos. En el puntaje global, los valores por debajo de 9 % se acompañaron de una mejor calidad de vida relacionada con la salud, mientras que, con aquellos mayores de 9 %, la calidad de vida se percibió como baja, hallazgo estadísticamente significativo (cuadro 2).

En cuanto al PedsQL 4.0™ (módulo general), se obtuvo una mejor percepción en los dominios escolar, físico y social, con valores promedio entre 74,4 y 88,5. El dominio emocional presentó un puntaje de 64,6 que lo cataloga como una percepción de calidad de vida deficiente para los niños encuestados sobre este dominio. En el puntaje global del PedsQL 4.0™, se obtuvo un valor promedio de 78,68 (cuadro 4).

**Cuadro 4:** Puntajes del PedsQL™-4.0, general

Dominio	N	Mínimo	Máximo	Promedio	DE
Escolar	25	45	90	74,4	14,81
Físico	25	59,38	100	88,5	13
Emocional	25	30	100	64,6	16,39
Social	25	55	100	87,2	14,58
Puntaje global	25	54,84	95	78,68	12,14

Por otra parte, para el módulo sobre diabetes (versión 3.2), se obtuvo una calidad de vida deficiente en los dominios de barreras y preocupación. Los dominios de cumplimiento y comunicación presentaron valores superiores a 70 e, incluso, el de comunicación fue el de la puntuación más alta. En cuanto al puntaje global del módulo sobre diabetes (versión 3.2), el valor es menor que el del PedsQL 4.0™ general; sin embargo, los dos se encontraron por encima de 70 (cuadro 5).

**Cuadro 5:** Puntajes del versión 3.2 PedsQL™, módulo sobre diabetes,

Dominio	N	Mínimo	Máximo	Promedio	DE
Síntomas	25	31,67	93,33	73,07	14,18
Barreras	25	15	100	60,20	21,96
Adherencia	25	50	100	80,83	15,87
Preocupación	25	0,00	100	54,33	33,08
Comunicación	25	18,75	100	83,75	22,75
Puntaje global	25	39,58	96,42	70,44	14,98

DE: desviación estándar

## Discusión

La diabetes mellitus es una de las enfermedades crónicas con mayor prevalencia en la población pediátrica y juvenil. La diabetes afecta muchos aspectos de la vida, no solo la salud, sino que impacta otros aspectos como el psicosocial, consecuentemente, se ve afectada la calidad de vida. Las medidas de calidad de vida relacionada con la salud han demostrado ser útiles para proporcionar una evaluación integral de la enfermedad y sus efectos en la vida diaria de los pacientes ^7^. El diagnóstico en edades cada vez más tempranas, plantea retos en la atención integral a los pacientes diabéticos y sus familias ^8^.

Se han realizado múltiples estudios a nivel global con el fin de caracterizar la calidad de vida relacionada con la diabetes. Uno de los más importantes fue el estudio TEENs, que abarcó una muestra global de 2.846 adolescentes de los cinco continentes y tuvo como objetivo caracterizar la calidad de vida relacionada con la diabetes mellitus de tipo 1. Se concluyó que la calidad de vida estaba significativamente relacionada con los valores de HbA1c, es decir, cuanto más bajos sean los valores de HbA1c, mejor es la calidad de vida.

Los resultados del presente estudio sugieren lo mismo, encontrándose un mejor puntaje de calidad de vida con los menores valores de hemoglobina glucosilada; también se observó que el acceso a los servicios de salud bien estructurados y el garantizar el seguimiento adecuado de estos pacientes, generaban mejores puntajes ^9^^).^

En el presente estudio, la mayoría de los adolescentes (grupo poblacional entre 12 y 18 años) tenía valores de HbA1c por encima de 7,5 % (rango: 5,5-12 %), similar a lo obtenido en el estudio TEENs, donde se encontraron valores entre 7,1 y 10,3 %. Cabe resaltar que los valores de HbA1c estaban por fuera de las metas de la *American Diabetes Association* (ADA) y la *International Society for Pediatric and Adolescent diabetes* (ISPAD), que los establecen en 7 % o menos como meta ideal para los pacientes diabéticos en tratamiento ^10^. Se considera, entonces, que los grupos poblacionales de adolescentes pueden presentar descompensaciones metabólicas, dado el amplio rango de hemoglobina glucosilada que manejan, así como ver afectados aspectos de la calidad de vida.

En el presente estudio, los adolescentes presentaron puntajes bajos en los dominios de barreras y preocupación, lo cual muestra la importancia del control metabólico para mejorar aspectos de la calidad de vida que puedan mejorar la percepción de estos pacientes.

Con respecto a otros comportamientos, como los estilos de vida, se puede observar que los puntajes totales más altos del PedsQL™ se relacionaron con valores más bajos de HbA1c, como lo demostrado en otros estudios publicados ^11^. Además, se presentaron puntajes altos en todos los dominios del módulo sobre diabetes mellitus de tipo 1 del PedsQL™, relacionados con un valor de hemoglobina glucosilada menor de 7 %, como lo evidenciado por Tahirovic, *et al.*^12^. Esto también se evidenció en el presente estudio, para valores menores de 9 %.

En algunos estudios, los dominios emocional y social del PedsQL 4.0™ (genérico o general) obtienen los puntajes más bajos, como también en algunos estudios de caso-control, en los cuales los puntajes menores en los dominios emocional, social y escolar correspondían a las cohortes diabéticas al compararlas con las cohortes saludables ^13^. En nuestra muestra, los puntajes más bajos fueron de los dominios emocional y escolar, con puntajes de 30 y 45 respectivamente, un hallazgo similar a los de otros estudios publicados ^14^.

En nuestros resultados, la percepción de calidad de vida comparada según el tipo de tratamiento se ve afectada negativamente con una relación estadísticamente significativa, entre los pacientes con múltiples inyecciones *versus* usuarios de la microinfusora (p<0,05). Estos hallazgos se correlacionan con lo evidenciado en un estudio del 2016 en México, publicado en la *Revista Médica del Instituto Mexicano del Seguro Social,* en el que se buscó analizar las indicaciones para el uso de terapia de infusión continua subcutánea de insulina en niños y adolescentes con diabetes mellitus de tipo 1; se encontró que las principales motivaciones que influyeron para su inicio fueron mejorar la calidad de vida de los pacientes y lograr un mejor control metabólico.

Anderson, *et al.,* en su trabajo publicado como parte del *Global TEENs Study* del 2017, refieren que uno de los tres comportamientos más relevantes para el control de la diabetes, que se relaciona significativamente con una mejor calidad de vida, es el monito reo diario de la glucosa en sangre. Por lo tanto, los pacientes con bomba de insulina y monito reo continuo de glucosa, como lo tienen los pacientes en Colombia, presentarían un mejor puntaje por esta razón ^15^. Esto también es sustentado en la literatura por Lukács, *et al.* (2018), quien comprobó que la bomba de insulina promueve una mejor calidad de vida relacionada con la salud ^16^.

Al comparar los tipos de tratamiento, se encuentra una actualización reportada en 2019, de Pala, *et al.*^17^, quienes llevaron acabo un metaanálisis para comparar la bomba de insulina frente a las inyecciones de insulina tradicionales (1 a 2 inyecciones diarias con esquemas de insulina cristalina y NPH). Además de valorar las posibles diferencias en el control metabólico y los episodios de hipoglucemia, en lo que se relacionó con la calidad de vida relacionada con la salud, todos los estudios se encontraron a favor de la elección de bombas de insulina en lugar de las múltiples dosis de insulina inyectadas, haciendo la claridad de que se necesitarían más estudios para definir mejor el perfil de los pacientes que se podrían beneficiar de la bomba de insulina *Vs.* los esquemas de inyecciones múltiples ^16^^,^^17^.

Como dificultades del estudio, trabajamos con un tamaño de muestra pequeño debido a que se llevó a cabo en plena pandemia por COVID-19, cuando se disminuyeron las consultas externas de las subespecialidades pediátricas y la mayoría de los pacientes se controlaban por telemedicina, una vez instauradas las medidas de prevención. En cuanto a la muestra, pudo existir un sesgo de selección, dado que ciertos niños que no acudieron a la cita o que no aceptaron participar en el estudio, pudieron ser aquellos que tenían un peor control metabólico.

En conclusión, se encontró una percepción de mejor calidad de vida en pacientes con bomba microinfusora que en aquellos con múltiples inyecciones, y en los que presentaban mejor valor de HbA1c. El puntaje global de la encuesta PedsQL™ general fue satisfactorio en promedio; sin embargo, en los dominios escolar y emocional, se encontraron los puntajes más bajos del estudio.

En cuanto a los resultados globales en calidad de vida percibida según la encuesta PedsQL™ 3.2 en nuestros pacientes con diabetes mellitus de tipo 1, se evidenció que es similar al a los de otros estudios publicados con mayor tamaño muestral. En nuestros pacientes, se encontraron diferencias estadísticamente significativas en aquellos con un mejor control metabólico (medido por el valor de HbA1c) así como en los pacientes en tratamiento con bomba microinfusora, quienes tenían mejores puntajes. Esto puede verse influenciado por un mejor cumplimiento del tratamiento o por un mayor número de controles y seguimiento médico similar a lo encontrado en la literatura científica.

Cabe resaltar las buenas puntuaciones en todos los grupos en el dominio comunicación, pudiendo esto estar relacionado con que este estudio se realizó en una población cautiva de dos centros de referencia de endocrinología pediátrica, donde el cuidado del diabético está bien estructurado.

A pesar de las limitaciones mencionadas, consideramos que los resultados del estudio aportan información relevante, no solo para la comunidad médica científica nacional, por ser el primer estudio relacionado en niños y adolescentes con diabetes de tipo 1 en el país, sino también, para la de la región.

Para finalizar, hay que reconocer las exigencias diarias que el tratamiento de la diabetes mellitus de tipo 1 supone para los pacientes y sus familias, y cómo esta interfiere en la calidad de vida. Por lo tanto, valorarla con escalas validadas como el PedsQL™ 3.2, módulo para diabetes, debería ser una parte más del manejo, con el fin de identificar de manera precoz posibles alteraciones y, así, poder establecer medidas de intervención individualizadas que permitan optimizar la atención y los resultados en nuestros niños y adolescentes con diabetes.
